# F39. MATERNAL IMMUNE ACTIVATION MODELS: MIND YOUR CAGING SYSTEMS!

**DOI:** 10.1093/schbul/sby017.570

**Published:** 2018-04-01

**Authors:** Flavia Müller, Ulrike Weber-Stadlbauer, Urs Meyer

**Affiliations:** Institute of Pharmacology and Toxicology, University of Zurich

## Abstract

**Background:**

Rodent models of maternal immune activation (MIA) are increasingly used as experimental tools to study neuronal and behavioral dysfunctions in relation to infection-mediated neurodevelopmental disorders such as schizophrenia and autism. One of the most widely used MIA models is based on gestational administration of poly(I:C) (= polyriboinosinic-polyribocytdilic acid), a synthetic analog of double-stranded RNA that induces a cytokine-associated viral-like acute phase response. The effects of poly(I:C)-induced MIA on phenotypic changes in the offspring are known to be influenced by various factors, including the precise prenatal timing, genetic background, and immune stimulus intensity. Thus far, however, it has been ignored whether differences in laboratory housing systems can similarly affect the outcomes of MIA models. Here, we examined this possibility by comparing poly(I:C)-based MIA in two housing systems that are commonly used in preclinical rodent research, namely the individually ventilated cage (IVC) and open cage (OC) systems.

**Methods:**

Pregnant C57BL6/N mice were kept in IVC or OC and treated with a low (1 mg/kg, i.v.) or high (5 mg/kg, i.v.) dose of poly(I:C), or with corresponding vehicle solution (pyrogen-free, sterile 0.9% NaCl; i.v.). MIA or control treatment was induced on gestation day (GD) 9 or 12, and the resulting offspring were raised and maintained in IVC or OC until adulthood for behavioral testing. An additional cohort of dams were used to assess the influence of the different caging systems on poly(I:C)-induced cytokine responses in the maternal plasma, placenta, and fetal brains 1 hr and 6 hrs post-treatment.

**Results:**

Maternal administration of poly(I:C) on GD9 caused a dose-dependent increase in spontaneous abortion in IVC but not in OC system, whereas MIA in IVC systems during a later gestational time-point (GD12) did not do so. Maternal and fetal pro-inflammatory cytokine responses to poly(I:C) were markedly higher in animals kept in IVC as compared to OC systems. The efficacy of MIA to induce long-term behavioral deficits was influenced by the different housing conditions, the dosing of poly(I:C), and the precise prenatal timing.

**Discussion:**

The present study identified the type of cage system as a novel factor that can confound the outcomes of MIA. Our findings thus urge the need to consider and report the kind of cages used in rodent MIA models. Providing this information seems pivotal to yield robust and reproducible results in these models.

